# Fabrication of High-Performance Densified Wood via High-Pressure Steam Treatment and Hot-Pressing

**DOI:** 10.3390/polym16070939

**Published:** 2024-03-29

**Authors:** Weizhi Huang, Yangxi Jin, Yi Guo, Jiaqi Deng, Haoyang Yu, Bobing He

**Affiliations:** College of Chemistry, Sichuan University, Chengdu 610064, China; 2020222030041@stu.scu.edu.cn (W.H.); 2021222030182@stu.scu.edu.cn (Y.J.); guoyi1@stu.scu.edu.cn (Y.G.); dengjiaqi@stu.scu.edu.cn (J.D.); yhy35218@stu.scu.edu.cn (H.Y.)

**Keywords:** wood material, sustainable structural material, top-down strategy, steam treatment

## Abstract

The fabrication of sustainable structural materials with high physical properties to replace engineering plastics is a major challenge for modern industry, and wood, as the most abundant sustainable natural raw material on the planet, has received a great deal of attention from researchers. Researchers have made efforts to enhance the physical properties of wood in order to replace plastics. However, it is also difficult to meet practical demands at a low cost. Herein, we report a simple and efficient top-down strategy to transform bulk natural basswood into a high-performance structural material. This three-step strategy involves partial removal of hemicellulose and lignin via treating basswood by boiling an aqueous mixture of NaOH and Na_2_SO_3_, and a high-pressure steam treatment (HPST) was applied to delignified wood followed by hot-pressing, which allowed the wood to absorb moisture uniformly and quickly. HPST-treated dense delignified wood (HDDW) has a tensile strength of ~420 MPa, which is 6.5 times better than natural basswood (~65 MPa). We systematically investigated the various factors affecting the tensile strength of this wood material and explored the reasons why these factors affect the tensile strength, as well as the intrinsic connection between the moisture absorbed through HPST and the increased tensile strength of HDDW. Through our experiments, we realized the enhancement mechanism of HDDW and the optimal experimental conditions for the fabrication of HDDW.

## 1. Introduction

As a synthetic material that can be used in a variety of applications, plastics play an important role in the modern materials industry. However, with the condition of the global environment becoming increasingly severe, non-degradable plastic waste on earth has begun to be a concern. Plastic products made in the past were designed for high durability, but now these plastics become a huge environmental hazard because of their durability. Therefore, in recent years, sustainable high-performance materials have attracted more and more interest [1,2,3,4]. Whether humans can shift their dependence on petroleum-based materials to renewable, biodegradable materials will determine the viability of sustainable development [5,6].

Natural wood is one of the most abundant sustainable materials on earth, and therefore wood-based materials have attracted significant interest in recent years. Wood-based materials have the advantage of being biodegradable, and processed wood-based materials can exhibit a variety of benefits. For example, wood materials that have been treated with delignification and densification can possess low density and high strength, and have the potential to replace plastic products in the future. However, the mechanical properties of natural wood are not good enough for advanced engineering applications because of its natural structural defects. The mechanical properties of structural materials determine their engineering applications [7]. As a result, natural wood is not able to meet modern society’s demands for structural materials and therefore requires a series of treatments to increase its mechanical properties.

Researchers have explored a variety of ways to produce high-performance cellulose-based materials, and we can divide these methods into two categories: top-down methods and bottom-up methods. The bottom-up methods are mainly intended to obtain cellulose nanofibers (CNF) with high crystallinity and high strength through mechanical or chemical defibrillation, and such high-performance CNFs can be assembled to fabricate larger-scale cellulose macrofibers [8]. However, such approaches generally need complex chemical and physical treatment, which consumes a large amount of time and energy [9,10].

By contrast, the top-down approach is to break natural raw materials (plants, wood, etc.) down to the appropriate dimensions, directly utilizing their existing hierarchical structure without further assembly [11]. Top-down approaches are easier to reach, low-cost, and more suitable for fabricating high-performance materials on a larger scale [12]. Densification is an important factor in improving the mechanical performance of wood-based materials in top-down approaches, and researchers have spent a great deal of time studying the factors affecting wood densification. In some studies around 2000, researchers focused on enhancing the mechanical properties of wood and improving its dimensional stability through thermal and mechanical treatments, and in some studies after 2010, researchers began to focus on further enhancing the mechanical properties of densified wood. These early studies emphasized the influence of external experimental conditions, such as temperature [13,14,15,16], pressure, heat treatment conditions, and vacuum [17], ignoring the influence of the internal microstructure and chemical composition of wood [18,19,20]. Meanwhile, the important role played by lignin in the wood densification process has only gradually been discovered in recent years.

In recent years, researchers have increasingly focused on modifying wood by partial/complete delignification, while preserving the hierarchical structure as well as the directionality of the cellulose fibers which benefit the materials [21]. The increased porosity due to delignification facilitates the functionalization of cellulose scaffolds and allows polymer matrices or other modifying agents to be inserted more easily into cellulose scaffolds. Various functionalized cellulose-based materials have been studied by researchers on this basis [22,23,24], such as transparent wood and hardened wood. Such treatments are usually followed by densification, in order to improve the mechanical properties [25]. Unfortunately, the structural impact of chemical delignification is not completely understood right now. In this study, we will focus on the top-down methods to fabricate high-performance wood materials.

The goal of this research is to enhance the tensile strength of hot-pressed wood through a simple and effective physical treatment and to investigate the intrinsic mechanisms by which this treatment enhances the tensile strength of wood materials. Herein, we report a low-cost, top-down strategy to fabricate high-performance wood materials using a three-step process involving chemical delignification, high-pressure steam treatment, and hot-pressing. Through a quick and simple high-pressure steam treatment, we have achieved a significant increase in the tensile strength of hot-pressed basswood of about 15%. We have also systematically investigated the various factors affecting the tensile strength of this wood material and explored the reasons why these factors affect its tensile strength. Through a large number of experiments, we finally screened out the best experimental conditions. The following is our fabrication approach for high-pressure steam-treated densified delignified wood (HDDW). In this work, we chose basswood as the raw material, which is a moderately hard, oil-containing, abrasion-resistant, and rot-resistant wood. Basswood has good toughness and workability, is not prone to cracking and deformation, and is excellent as a wood raw material for hot-pressing. We used NaOH/Na_2_SO_3_ solution as a delignification agent to partly remove lignin and hemicellulose in raw natural basswood, and an autoclave was used to disperse moisture into the wood uniformly and quickly. Finally, after the high-pressure steam treatment, the delignified wood was densified by hot-pressing. This cost-effective and simple approach provides an efficient way to fabricate high-performance sustainable materials without the addition of any non-degradable components.

## 2. Materials and Methods

### 2.1. Materials

Basswood blocks (100 × 40 × 5 mm^3^) were purchased from Shanghai Mingteng Co., Ltd. (Shanghai, China). The chemicals used in delignification were sodium hydroxide (>98%), sodium sulfite (>98%), and deionized water, which were purchased from Chengdu Kelong Chemicals Co., Ltd. (Chengdu, Sichuan, China). The autoclave which was used for high-pressure steam treatment was purchased from Guangdong Midea Electric Appliances Co., Ltd. (Guangzhou, Guangdong, China). The densification of wood was completed by a SY-6210-A plate vulcanizing press machine purchased from Shiyan Precision Instruments Co., Ltd. (Dongguan, Guangdong, China). The chemicals used for wood components determination were ethanol, toluene, and sulfuric acid, which were purchased from Chengdu Kelong Chemicals Co., Ltd.

### 2.2. Methods

**Figure 1:**
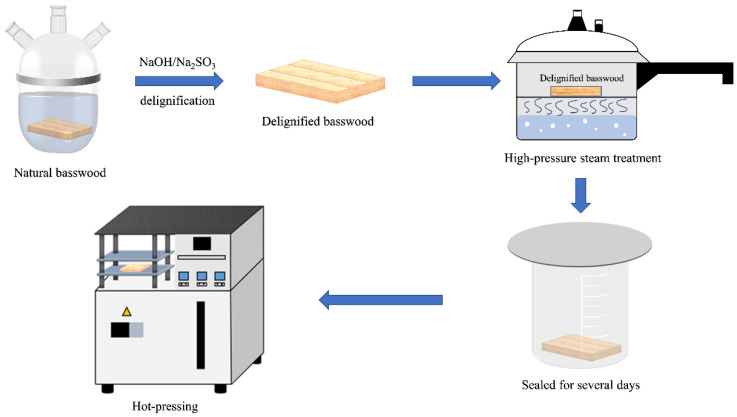
Fabrication approach of high-pressure steam-treated densified delignified wood (HDDW).

The complete fabrication process is shown in Figure 1.

#### 2.2.1. Chemical Delignification Procedure

Basswood samples were cut to the dimensions 100 × 40 × 5 mm^3^ (longitudinal × tangential × radial). The untreated wood (UW) samples were stored at 20 °C/65% relative humidity for 1 week before following treatment. An aqueous solution of 2.5 M NaOH and 0.4 M Na_2_SO_3_ was prepared, and the basswood blocks were immersed in the mixed solution at 110 °C for 0–10 h in order to partially remove hemicellulose and lignin. After delignification, the basswood blocks were washed in boiling deionized water with an exchange of water 1 time per hour until the pH value of the water became 7.0. The washed wood blocks were dried under 105 °C for 1 day.

#### 2.2.2. High-Pressure Steam Treatment (HPST)

The dried wood blocks were placed in an autoclave and treated with high-pressure steam for several minutes (the specific time depended on the target moisture content), and the moisture content of the wood blocks was controlled by the duration of the high-pressure steam treatment (HPST). The longer the HPST lasts, the more moisture is absorbed by the wood. The moisture content of the wood blocks after HPST can be calculated by weighing the wood blocks. Once the target moisture content was reached, the blocks were removed from the autoclave and sealed in an airtight container for 2 days so that the water absorbed by the blocks could be dispersed uniformly.

#### 2.2.3. Densification Procedure

The basswood blocks with 0–50% moisture content were firstly pressed at 110 °C under a pressure of 2 MPa for 1 h so that excessive damage to cellulose fibers was avoided, and then the pressure was increased to 5–20 MPa, and hot-pressing was continued for 24 h. After densification, we obtained high-pressure steam-treated densified delignified wood (HDDW). We also hot-pressed delignified basswood without HPST as a control group, which is named densified delignified wood (DDW).

### 2.3. Measurements

#### 2.3.1. Tensile Strength

Only the center portion of each HDDW sample was removed for subsequent processing, as uneven compression of the edge areas can lead to inaccurate tensile strength. The center portion of the removed HDDW was divided into three parts, and these specimens were cut by a CZ-1007 dumbbell prototype machine (Yangzhou Changzhe Testing Machinery Co., Ltd., Yangzhou, Jiangsu, China) before testing. Samples damaged during the cutting process will be discarded. The tensile properties were tested according to ISO 527 standard [26] specification using a LY-1066 A universal testing machine (Guangdong Liyi Technology Co., Ltd., Dongguan, Guangdong, China) with a testing speed of 5 mm/min and a maximum load of 20 kN. Every wood specimen was stored in a drying apparatus before the tensile test in order to avoid absorbing moisture. Three samples from the same HDDW were tested for parallel experiments, and three different HDDWs fabricated under the same experimental conditions were tested for replicate experiments. The raw materials used for the preparation of these HDDWs were taken from the same part of the basswood.

#### 2.3.2. Scanning Electron Microscopy Analysis (SEM)

An Apreo-S-HiVoc scanning electron microscope (SEM) purchased from Thermo Fisher Scientific Co., Ltd. (Shanghai, China) was used for observing the microstructure of NW and HDDW samples. These samples were sputter-coated with gold for about 30 s to increase their conductivity before the SEM analysis.

#### 2.3.3. X-ray Diffraction (XRD)

An X-ray diffractometer (Ultima IV, Rigaku Corporation, Tokyo, Japan) was used to detect the degree of crystallinity of cellulose within the wood samples using Cu-Kαradiation. The scan was executed at 40 kV and 30 mA current. The scan range was set to 5–40° and the scan speed was 2°/min. The obtained X-ray spectra were peak-fitted using Origin (Version 8) software applying the Gaussian function, and the crystallinity index was calculated using the following equation:

(1) $$CrI=\frac{I_{200}-I_{am}}{I_{200}}\times 100\%$$

*CrI* is the crystallinity index of cellulose, *I*_200_ is the intensity of the peak that is around 2*θ* = 30°, and *I_am_* is the minimum intensity between a peak of 200 and a peak of 110, which is around 2*θ* = 18°.

The crystal size of cellulose was calculated by the Scherrer equation:

(2) $$D_{hkl}=\frac{K\lambda }{cos\theta \sqrt{B_{hkl}^{2}-b^{2}}}$$

or

(3) $$D_{hkl}=\frac{K\lambda }{\beta cos\theta }$$

*K* is the Scherrer constant, *λ* is the X-ray wavelength, *B_hkl_* and *β* are the half-peak full widths of the *hkl* peak, *b* is the instrumental broadening, and 2*θ* is the diffraction angle [27].

#### 2.3.4. Determination of Lignin, Cellulose, and Hemicellulose Contents

1. Lignin

A filter paper bag containing wood samples (the mass was *m*_0_) was placed in a Soxhlet extraction apparatus. The samples were extracted with 95% ethanol for 4 h and then with an ethanol–toluene solution (1000 mL:427 mL) for 8 h. A dropper was used to remove as much solvent as possible, and then the samples were washed with 50 mL of ethanol to remove residual toluene. The filter paper bag was transferred to a beaker and extracted sequentially with three portions of 500 mL deionized water at 100 °C, changing the deionized water every hour. Then, the filter paper bag was washed with 50 mL of ethanol and the samples were left in the beaker and dried in air inside the paper bag.

The air-dried samples were transferred to a small beaker with a glass lid and 15 mL of H_2_SO_4_ (72%) was added into the samples slowly while stirring. The samples and acid were stirred continuously for at least 1 min to ensure they were mixed evenly. Then, stirring continued for 2 h at 20 °C in a water bath. After this, the sulfuric acid concentration was diluted to 3% by adding 560 mL of deionized water to the solution, and the solution was boiled for 4 h. A reflux condenser was used in the process.

After the insoluble components precipitated, the residual acid was washed off with 500 mL of hot water, and the insoluble components were filtered out again. The samples with filter paper were dried in an oven at 105 °C until the mass was constant (*m_c_*). The dry mass of the quantitative filter paper (*m_f_*) had been measured in advance.

The lignin content ($\omega_{l}$) was calculated as:

(4) $$\omega_{l}=\frac{m_{c}-m_{f}}{m_{0}}\times 100\%$$

2. Cellulose and Hemicellulose

A wood fiber sample (2 g, *m*_1_), sodium chlorite (0.6 g), acetic acid (0.5 mL), and deionized water (65 mL) were added to a 100 mL three-necked flask and water-bathed at 75 °C for 1 h. The solution was then removed with a syringe, leaving the wood fibers in the flask. This step was repeated three times until the wood fibers turned completely white. The resulting white wood fibers were rinsed several times with deionized water and then dried in an oven at 120 °C. The fiber mass after drying (*m*_2_) is the sum of cellulose and hemicellulose mass.

The dried fibers were soaked in 10% sodium hydroxide solution (1 g fibers: 20 mL NaOH solution) for 16 h at room temperature to hydrolyze hemicellulose. The remaining fibers were rinsed several times with deionized water until the pH was close to 7, and then dried in an oven at 120 °C. The mass of dried fibers was m_3_ (i.e., the mass of cellulose).

The cellulose content ($\omega_{c}$) was calculated as:

(5) $$\omega_{c}=\frac{m_{3}}{m_{1}}\times 100\%$$

The hemicellulose content ($\omega_{h}$) was calculated as:

(6) $$\omega_{h}=\frac{m_{2}-m_{3}}{m_{1}}\times 100\%$$

## 3. Results and Discussion

In our experiments, we found several factors that had a large effect on the tensile strength of hot-pressed wood. Through control tests, we screened the best experimental parameters for treating wood samples in the HDDW samples. The following outlines the screening process of the optimal experimental conditions and the exploration process of the mechanisms by which these conditions affect the tensile strength of HDDW.

### 3.1. Effect of Delignification

In recent studies, lignin has been recognized as an important factor influencing the mechanical properties of hot-pressed wood. Since cellulose, hemicellulose, and lignin have different stabilities in chemical solutions [28], we used a sodium hydroxide/sodium sulfite mixture to remove lignin, which partly removes lignin and hemicellulose while retaining most of the cellulose.

**Figure 2:**
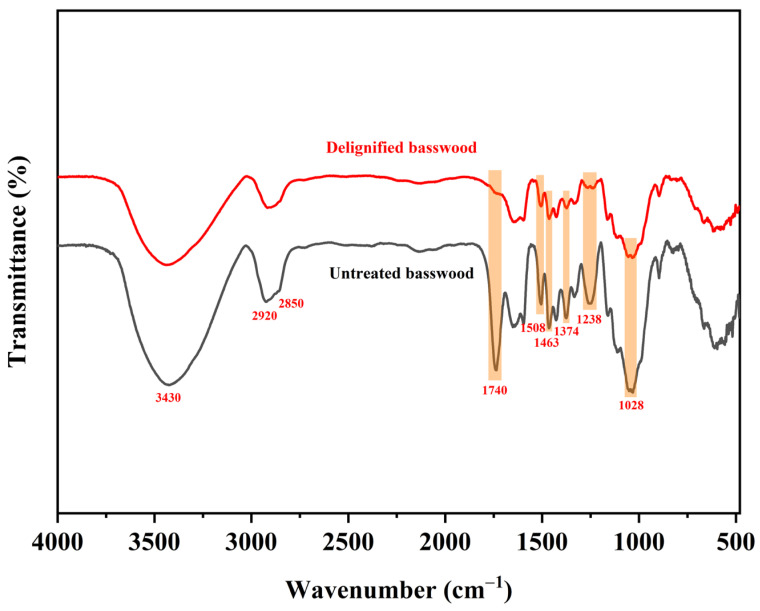
FTIR spectrum of untreated basswood and delignified basswood.

FTIR spectroscopy was used to verify the differences in the chemical stability of cellulose, hemicellulose, and lignin. The FTIR spectrums of untreated and delignified basswood are shown in Figure 2. The bands associated with these three chemicals are labeled and summarized in Table 1. Absorption bands with significant differences are labeled as orange areas in Figure 2. The absorption bands appearing at 3430 cm^−1^ and 2920 cm^−1^ were assigned to the –OH and C–H stretching vibrations of cellulose. The characteristic bands at 1508 cm^−1^, 1463 cm^−1^ and 1028 cm^−1^ corresponded to the C=C aromatic ring stretching vibration [29], –CH_2_ bending vibration, and C–O stretching vibration of lignin, respectively. The absorption bands at 2850 cm^−1^, 1740 cm^−1^, and 1238 cm^−1^ corresponded to the C–H stretching vibration, C=O stretching vibration, and C–O stretching vibration of hemicellulose, respectively [30]. The characteristic band at 1374 cm^−1^ corresponded to the CH bending vibration of cellulose and hemicellulose. As can be seen from the figure, the absorption bands at 1740 cm^−1^ and 1238 cm^−1^ almost completely disappeared after delignification, and the intensity of the absorption bands at 1374 cm^−1^ decreased significantly, which corresponded to the C=O stretching vibration, C–O stretching vibration, and CH bending vibration of hemicellulose, implying that the content of hemicellulose decreased dramatically after delignification. This result is also consistent with our subsequent content determination. The intensity of the absorption bands at 1508 cm^−1^, 1463 cm^−1^, and 1028 cm^−1^ all decreased, which is consistent with the result that lignin was partly removed. The absorption bands corresponding to cellulose did not change significantly, and cellulose had the best stability in this mixed solution, as evidenced by the subsequent test results.

**Figure 3:**
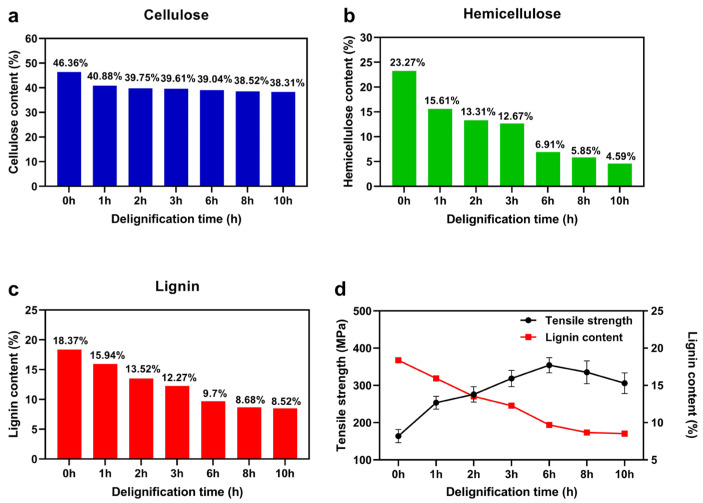
Characterization of wood components and the tensile strength of HDDW with different delignification times. (**a**) Variations in cellulose content with delignification duration. (**b**) Variations in hemicellulose content with delignification duration. (**c**) Variations in lignin content with delignification duration. (**d**) The tensile strength of HDDW with different delignification times.

In addition to FTIR, we also determined the change in their content after immersion in a boiling hydroxide/sodium sulfite mixture. Figure 3 shows the change in the content of cellulose, hemicellulose, and lignin after immersion in a boiling hydroxide/sodium sulfite mixture. It can clearly be seen that cellulose mostly remained, while hemicellulose and lignin were removed in larger amounts. As the immersion time increased, hemicellulose was almost completely removed, lignin was removed by more than half, and the cellulose content remained almost stable after a small early decrease. The results are in accordance with the FTIR results.

After these tests, we began to control the lignin content in basswood by adjusting the delignification time and exploring its effect on the mechanical performance of hot-pressed delignified basswood. Figure 3d shows the tensile strength of HDDW with different delignification durations after hot-pressing. The results show that the partial removal of lignin can effectively increase the strength of hot-pressed wood. Without lignin removal, it is difficult for untreated wood to be pressed densely [31], and many voids left between the cellulose fibers can be seen (this is described in detail later in the section “Morphological Analysis”). However, the tensile strength of HDDW decreased as the delignification duration was further increased. This may be due to the fact that excessive lignin removal causes the lack of lignin binders between cellulose fibers and these cellulose fibers become fragile during hot-pressing [32]. Some studies have shown that excessive delignification time leads to a decrease in the degree of polymerization of cellulose [33], which also reduces the excellent mechanical properties of the cellulose fiber itself. This is also an important reason for the decrease in the tensile strength of HDDW with increasing delignification time.

**Figure 4:**
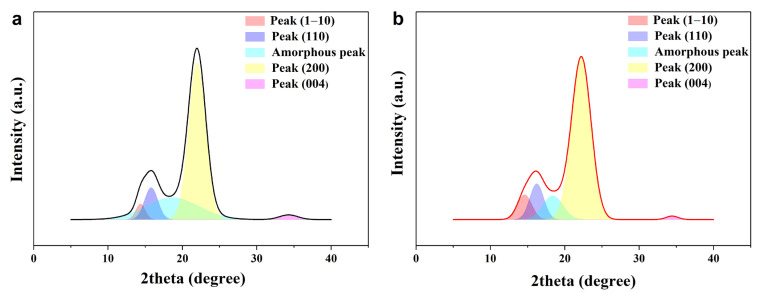
XRD analysis of untreated basswood and delignified basswood. (**a**) X-ray diffractogram of untreated basswood. The amorphous peak area of untreated basswood has a large percentage. (**b**) X-ray diffractogram of delignified basswood. The peak area in the amorphous region is significantly reduced.

Figure 4 is the X-ray diffractogram of untreated basswood and delignified wood. The XRD spectrum shows the changes in cellulose crystallinity after delignification. Untreated basswood has more amorphous regions and the amorphous peak area has a larger percentage. After delignification, lignin and hemicellulose, which exist in amorphous regions, are partly removed. This allows the rearrangement of cellulose in the amorphous region and also leads to an increase in the crystallinity of cellulose [34]. The cellulose crystallinity index calculated by Equation (1) is also in accordance with this conclusion, as shown in Table 2. The cellulose crystallinity of untreated basswood is 86.02 ± 0.64%, while the cellulose crystallinity of delignified basswood is 88.36 ± 0.56%.

The crystal size calculated by Equation (3) is also shown in Table 2. The crystal size of the peak (200) of untreated basswood is 30.37 ± 0.32 Å and the crystal size of the peak (200) of delignified basswood is 26.22 ± 0.54 Å. There is a decrease in the crystal size of cellulose after delignification, which also indicates an increase in the degree of crystallinity, a conclusion that is in accordance with the above results. All results suggest that the crystallinity of cellulose in basswood does increase after delignification.

The increase in cellulose crystallinity also reveals the reason for the high tensile strength of HDDW. The cellulose crystallinity did not decrease but increased after delignification, which indicates that the regular alignment of cellulose was not affected, and CNFs with excellent properties underwent rearrangement in the amorphous regions and densification, leading to the high performance of HDDW.

### 3.2. Effect of Pressure in Hot-Pressing

**Figure 5:**
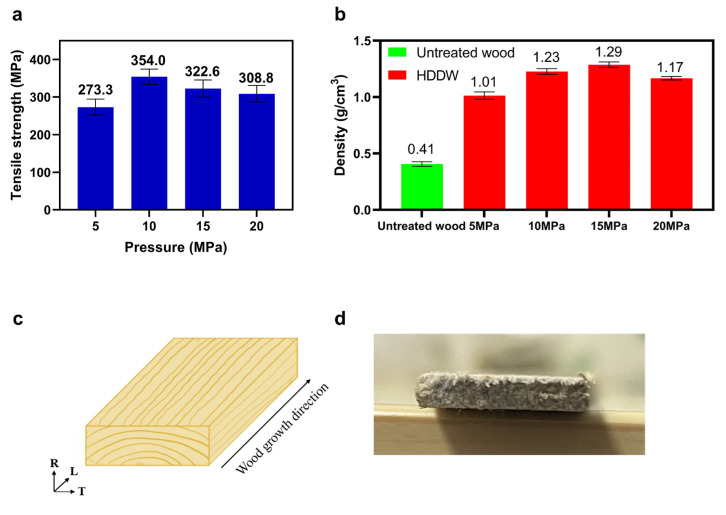
Effect of pressure in hot-pressing on the tensile strength and densities of HDDW. (**a**) The tensile strength of DDW with different pressures in hot-pressing. (**b**) Comparison of the densities of NW and HDDW with different pressures in hot-pressing. (**c**) R-T-L division of basswood. (**d**) Photograph of the R-T plane of HDDW (group of 15 MPa).

During hot-pressing, the pressure also has an effect on the degree of densification as well as the tensile strength of HDDW [35,36,37]. We tested the tensile strength of HDDW under different hot-pressing pressures under the conditions of delignification duration of 6 h and moisture content of 10%, and the results are shown in Figure 5. When the hot-pressing pressure was 5 MPa, the HDDW had the lowest tensile strength of the four groups. When the pressure was increased to 10 MPa, there was a large increase in the tensile strength, and the 10 MPa group had the highest tensile strength among these four groups. This is due to the fact that higher pressure reduces the interfacial distance between cellulose nanofibers, which facilitates the formation of more hydrogen bonds. With a further increase in pressure, the tensile strength of HDDW instead starts to decrease gradually. This may be related to the ability of cellulose fibers to withstand the pressure. In a high-temperature and high-pressure environment, the water inside the wood fibers vaporizes rapidly, and this rapid vaporization will produce counter pressure between cellulose fibers, which we have already mentioned in the previous section. Excessive pressure leads to higher counter pressure, which facilitates the destruction and fracture of cellulose fibers. As a result, the tensile strength of HDDW decreased when the pressure exceeded 10 MPa.

We also tested the densities of HDDW obtained by hot-pressing delignified wood blocks at different pressures and compared them with untreated basswood. As can be seen from the figure, the trend of the density of HDDW and tensile strength is not exactly the same. HDDW has the highest tensile strength when the pressure is 10 MPa. However, HDDW has the highest density when the pressure is 15 MPa, and the two are not maximized at the same pressure. From the data in the figure, it can be concluded that a higher density does not necessarily mean a higher tensile strength for HDDW. The density indicates the degree of densification of HDDW, and this conclusion shows that there is no absolute correlation between the tensile strength and the degree of densification. In most cases, a higher density always means that the cellulose molecules are more tightly aligned with each other, which contributes to more hydrogen bonding. However, the tensile strength is not consistent with that conclusion. Figure 5d shows the R-T plane morphology of HDDW of the 15 MPa group, and some exposed wood fibers can be seen on the R-T plane. In our opinion, excessive pressure destroyed the structure of the cellulose fibers, which led to the extrusion of some cellulose fibers after breakage during the hot-pressing process. Although the density of HDDW was increased in this process, the destruction of the fibers led to a decrease in tensile strength instead.

### 3.3. Effect of HPST and Moisture Content

**Figure 6:**
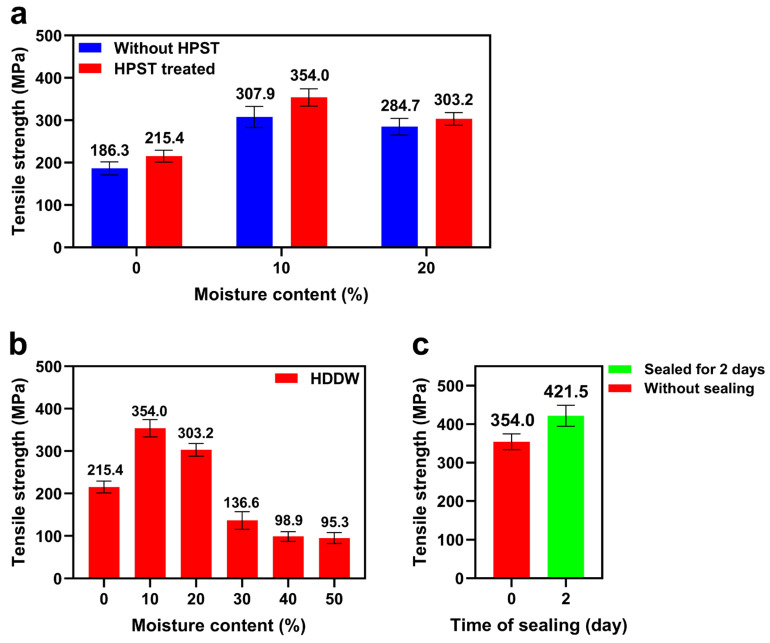
Effect of HPST and moisture content on the tensile strength of HDDW. (**a**) Comparison of the tensile strength of DDW and HDDW. The tensile strength of HDDW was significantly higher than that of DDW under the same experimental conditions. (**b**) The tensile strength of HDDW with different moisture content before hot-pressing. (**c**) Comparison of the tensile strength of HDDW and HDDW that was sealed for 2 days before hot-pressing.

Some researchers have reported the effect of steam treatment on the properties of hot-pressed wood [38,39]. To verify the effectiveness of HPST and systematically study its effect, we compared the tensile strength of DDW and HDDW. Two groups were hot-pressed under the same conditions. The moisture content of the wood blocks before hot-pressing was controlled at 0–20%. Dried delignified wood blocks were placed in an autoclave and treated with HPST for a short length of time, and the moisture content of the wood blocks was controlled by the duration of the HPST. The DDW group was stored for 1–10 days at 25 °C and 95% relative humidity before hot-pressing to achieve the same moisture content. The results are shown in Figure 6.

Figure 6a shows that the HPST resulted in a significant increase in the tensile strength of DDW in the moisture content range of 0–20%. Compared to natural water absorption, wood blocks can quickly absorb moisture to reach the target moisture content during the HPST process. High-temperature, high-pressure water vapor can infiltrate the wood blocks more easily, allowing the interior area of the delignified wood block to absorb moisture as well. This leads to a more uniform absorption of moisture during the HPST process compared to natural moisture absorption.

Moisture content is also an important factor affecting the tensile strength of hot-pressed wood. Figure 6b shows the tensile strength of HDDW with different moisture contents before hot-pressing (delignification time was uniformly 6 h). It can be seen that a small amount of moisture leads to an increase in the tensile strength of the HDDW, while too high a moisture content leads to a decrease in the tensile strength instead. The results show that the group with 10% moisture content has the best tensile strength. The tensile strength of wood with different moisture contents differed greatly after hot-pressing. This is because the H_2_O molecules inside the wood lumina are able to form hydrogen bonds with the hydroxyl groups on multiple cellulose macromolecules during hot-pressing, which helps to increase the interfacial area between the cellulose fibers [40]. Due to the presence of a large number of hydroxyl groups in the cellulose molecules, a larger interfacial area means more hydrogen bonds between the cellulose fibers, which increases the difficulty of the relative sliding between wood cell walls. HDDW undergoes relative sliding between collapsed wood cell walls during the process of tensile failure, and the wood cell walls are pulled out and destroyed along the fracture surfaces [41], so the increasing difficulty of relative sliding between cell walls leads to a higher tensile strength.

Also, the H_2_O molecules can act as plasticizers for the cellulose fibers, increasing the flexibility of the cellulose fibers. These plasticizers allow cell walls and the internal cellulose fibers to densely pack together [42]. As a result, delignified wood with an extremely low moisture content has a smaller interfacial area after hot-pressing, leading to a lower tensile strength. However, it is interesting that excessive moisture content leads to a substantial decline in the tensile strength of the HDDW. In our opinion, this result is related to the fiber saturation point of wood, which means the maximum water-absorbing capacity of wood cell walls. Water in wood exists in three main forms: free water, adsorbed water, and bound water [43]. Free water and adsorbed water account for the majority of wood moisture. When the moisture content of wood is below the fiber saturation point, almost all of the moisture in wood exists in the form of adsorbed water, and there is no free water in the cell cavities. When the moisture content of wood exceeds the fiber saturation point, the excess water exists as free water in the cell cavities and cellular interstitial space. The free water will vaporize rapidly in a high-temperature and high-pressure environment, and the counter pressure generated by this rapid vaporization process may lead to deformation and fracture of cellulose fibers, resulting in an extremely low tensile strength (even lower than the 0% moisture group).

**Figure 7:**
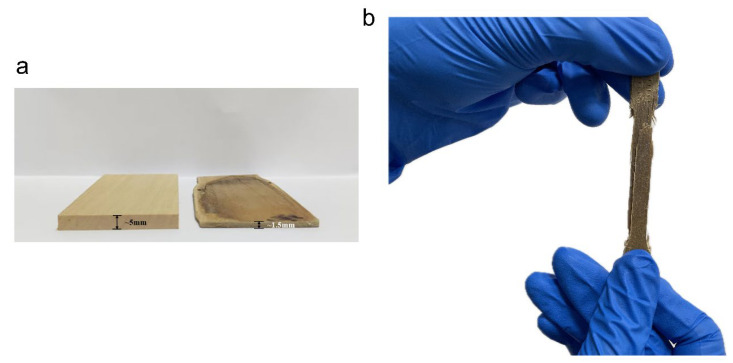
Photographs of HDDW. (**a**) Comparison of untreated basswood and HDDW. The thickness of HDDW is about 30% of that of untreated basswood. (**b**) Photograph of a tensile fractured HDDW sample.

In subsequent experiments, we found that the tensile strength of HDDW was further increased if the wood was left in an airtight container after the HPST (to ensure that the moisture content remained unchanged) for 2 days before hot-pressing, as shown in Figure 6c. These wood blocks were hot-pressed at the same moisture content (10%); however, the samples that were not left in the airtight container for a few days after HPST had a lower tensile strength after hot-pressing. We believe that the reason why sealing the blocks for several days resulted in an increase in the tensile strength of the HDDW was that the moisture absorbed by the blocks via HPST was not completely uniform. Wood can absorb a lot of moisture in a short period of time under high-pressure steam, but due to the block shape of the wood, the moisture content in the center of the wood is not as high as on the surface of the wood. This may result in the moisture content in the center of the wood being lower than the target value, and the moisture content of the wood surface may have exceeded the fiber saturation point, so some water may be present as free water in the fibers on the surface. Such small amounts of free water contribute very little to the densification of the wood during hot-pressing, and may even be detrimental to the wood fibers (as mentioned earlier). After being placed in an airtight container for several days, the moisture absorbed by the wood through the HPST diffused through the concentration gradient to the center of the wood, and the moisture content of the fibers in all parts of the wood became uniform. Uniform moisture leads to the generation of more hydrogen bonds and high tensile strength. Figure 7b shows the fracture morphology of HDDW splines. The splines split into two layers from the center after the tensile fracture occurred, leaving a flat fracture surface, which evidences that the fracture mechanism of HDDW is mainly interlayer relative sliding. Due to the excellent tensile strength of cellulose fibers along the fiber direction, the interlayer relative sliding precedes the fiber fracture during the process of tensile fracture of the spline. The presence of water molecules, as mentioned before, increases the difficulty of interlayer relative sliding of cellulose, and since the fracture mechanism of HDDW is dominated by interlayer relative sliding, this also implies an increase in tensile strength.

### 3.4. Morphological Analysis

**Figure 8:**
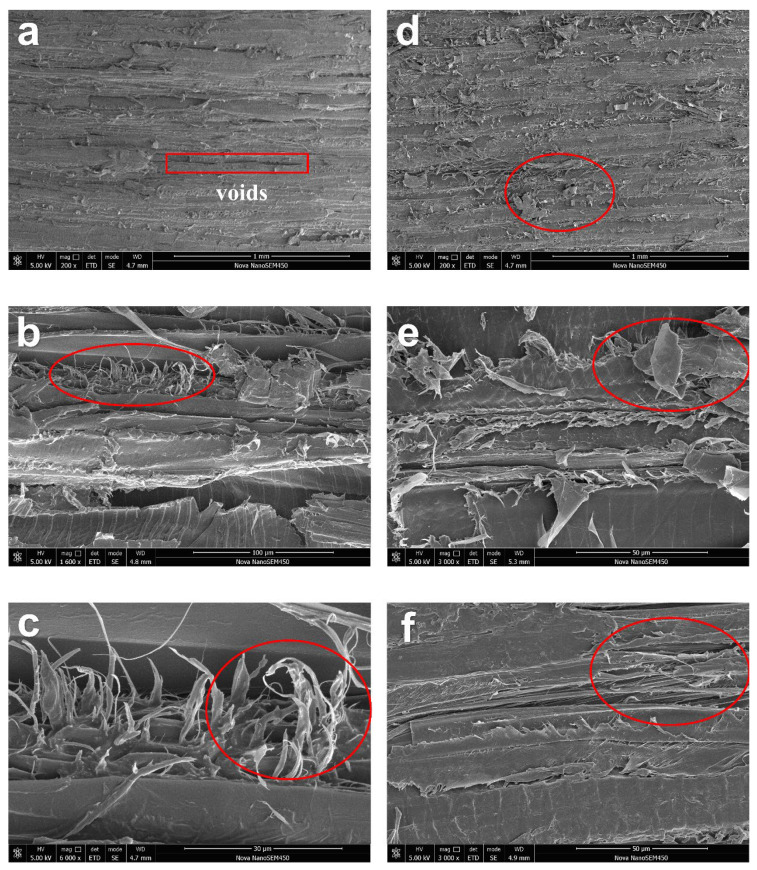
SEM images of the fracture surface of hot-pressed untreated basswood and HDDW. Fibers with characteristic morphology have been circled in red areas. (**a**) Microscopic morphology of hot-pressed untreated basswood. (**b**) SEM image of pulled-out fibers along the fracture surface in a filamentous form. (**c**) SEM image of fractured filamentous fibers. (**d**) Microscopic morphology of HDDW. There is almost no void visible on the fracture surface. (**e**) SEM image of pulled-out fibers in sheets along the fracture surface. Few filamentous fibers are visible. (**f**) SEM image of tightly bonded fibers that are in the form of bundles inside the fracture surface.

Figure 8a–c shows the tensile fracture surface of hot-pressed untreated basswood. At a smaller magnification, a few fibers that were pulled out from the fracture surface can be seen, and after magnifying, the fibers pulled out along the fracture surface in a filamentous form become visible. It is obvious that these fibers are not tightly entangled with each other (Figure 8b). Since the untreated basswood was not delignified, the wood lumina was not fully compressed, and a large number of voids existed between the folded wood cell walls (Figure 8a). During the tensile process, the tensile fracture was initiated by relative sliding among opening wood lumina, and they were pulled out one by one and fractured. Due to the existence of voids between the cell walls, cellulose fibers interacted weakly with each other, which resulted in the fibers appearing as filaments after the tensile fracture occurred, and they were not able to be closely bonded together to resist the tensile stresses. These fractured filamentary fibers can be found at various locations along the bundle of cellulose fibers on the fracture surface (Figure 8c), and the breakage of such relatively independent fibers during the relative sliding process leads to the fracture of a hot-pressed NW sample.

Figure 8d–f shows the tensile fracture surface of HDDW. As can be seen at a smaller magnification, there are more fibers pulled out along the fracture surface of HDDW, and the pulled-out fibers are in sheets, which is different from the hot-pressed untreated basswood (Figure 8d). At a size of 50 microns, the fractured fibers are tightly intertwined, so that we seldom see filamentous fibers on the fracture surface, and the vast majority of them appear as sheets. After delignification, the wood cell walls became porous, and the porous cell walls collapsed completely during hot-pressing. Collapsed wood cell walls were intertwined with each other densely (Figure 8e), and the cellulose fibers were tightly aligned. During the process of tensile fracture of HDDW, relative sliding occurred among the collapsed wood cell walls, and such relative sliding required more energy due to the presence of a large number of hydrogen bonds among the cellulose molecular chains. When tensile fracture occurred, the dense wood cell walls were pulled out from the fracture surface and the fibers inside the HDDW were exposed to the fracture surface. The fibers inside the fracture which are tightly wound are in the form of bundles (Figure 8f), so these fibers are more resistant to tensile stresses. The figures show that the fibers of HDDW have strong interactions with each other. The strong interactions between fibers make it more difficult for the wood cell walls to be pulled out. As a result, HDDW has a higher tensile strength than hot-pressed untreated basswood.

### 3.5. Statistical Analysis

**Figure 9:**
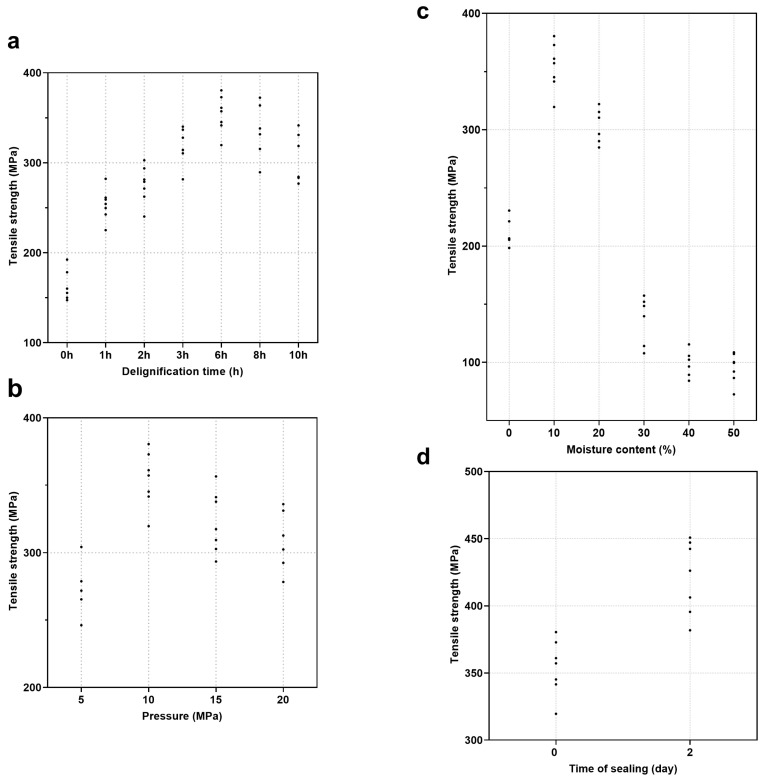
Values of tensile strength of HDDW fabricated under different experimental conditions. (**a**) Delignification time. (**b**) Pressure in hot-pressing. (**c**) Moisture content. (**d**) Time of sealing.

The tensile strengths of HDDW fabricated under different experimental conditions are shown in Figure 9. The one-way ANOVA results employed to verify the significance of these experimental factors affecting the tensile strength of HDDW are shown in Table 3, Table 4, Table 5 and Table 6. Table 3, Table 4, Table 5 and Table 6 present the *p*-values for these factors, which determine the influence of these factors on the tensile strength of HDDW. Statistical significance was set at *p <* 0.05. There is a significant correlation between the factor and the tensile strength of HDDW when *p <* 0.05. In contrast, a *p*-value that is equal to or higher than 0.05 means the absence of correlation between the factor and the results.

Table 3, Table 4, Table 5 and Table 6 show that the four factors (delignification time, pressure, moisture content, and sealing time) all satisfy the conditions of *p* < 0.05 and F > F critical. These results demonstrate that the influence of these four factors on the tensile strength of HDDW is statistically significant.

### 3.6. Summary of Enhancement Mechanisms

From the above experiments, it can be inferred that the high tensile strength of HDDW mainly comes from the presence of a large number of hydrogen bonds between cellulose fibers. Partial removal of lignin from untreated basswood allows the wood cell walls to become porous, and the porous cell walls are completely collapsed by hot-pressing, allowing the cellulose fibers to be tightly entangled. The shortened interfacial distances between the cellulose lead to more hydrogen bonding, which becomes a central factor in the enhancement of the tensile strength of the hot-pressed wood. HPST allows the wood to absorb water quickly and uniformly. An appropriate amount of moisture can then form hydrogen bonds with multiple hydroxyl groups on the cellulose, a process that also shortens the interfacial distances between the cellulose nanofibers, which enhances the HDDW based on the same mechanism as an appropriate pressure.

## 4. Conclusions

In summary, we report a simple top-down strategy to fabricate high-pressure steam-treated densified delignified wood (HDDW). The three-step process involves chemical delignification, high-pressure steam treatment, and hot-pressing. The tensile strength of HDDW was significantly enhanced because of the shortened interfacial distance between cellulose fibers and increased hydrogen bonds. The physical and mechanical properties of HDDW were evaluated through tensile tests and density tests. The chemical properties of HDDW were analyzed through FTIR and XRD. Microscopic morphology was observed through SEM, and a statistical analysis analyzing the average results of tensile tests was conducted. The following can be concluded based on the obtained results:

1. FTIR analysis and determination of lignin, cellulose, and hemicellulose showed that lignin and hemicellulose were partly removed from basswood after the delignification treatment.
2. XRD analysis revealed an increase in the crystallinity of cellulose and a decrease in crystal size after delignification treatment, which indicated that the regular alignment of cellulose was not affected by delignification.
3. Removal of lignin made the wood cell wall porous, which had an effect on the tensile strength of HDDW. Moderate removal of lignin resulted in a significant increase in the tensile strength of HDDW, while excessive removal of lignin resulted in a decrease in the tensile strength of HDDW.
4. Higher pressure in hot-pressing enhanced the tensile strength of HDDW by shortening the interfacial distance between cellulose fibers, which also changed the number of hydrogen bonds between cellulose fibers. However, excessive pressure led to higher counter pressure from water, which resulted in the fracture of cellulose fibers and a decrease in the tensile strength of HDDW.
5. Moisture absorbed by HDDW through HPST could form hydrogen bonds with hydroxyl groups on multiple cellulose molecules during the hot-pressing process, which led to a shorter interfacial distance between cellulose fibers and more hydrogen bonds. Excessive moisture also resulted in higher counter pressure and a decrease in the tensile strength of HDDW.
6. The microscopic morphology observed by SEM was consistent with the above conclusions. The cellulose fibers of HDDW were more tightly bonded compared to hot-pressed basswood, which was the source of the high tensile strength of HDDW.

## Figures and Tables

**Table 1:** Characteristic bands and associated functional groups in FTIR spectrum.

**Wavenumber (cm^−1^)**	**Functional Group**	**Corresponding Compound**
3430	Stretching vibration of OH	Cellulose
2920	Stretching vibration of CH	Cellulose
2850	Stretching vibration of CH_2_	Hemicellulose
1740	Stretching vibration of C=O	Hemicellulose
1508	Aromatic symmetrical stretching vibration of C=C	Lignin
1463 1374	Bending vibration of CH_2_ Bending vibration of CH	Lignin Cellulose, hemicellulose
1238	Stretching vibration of C-O in C-C=O	Hemicellulose
1028	Stretching vibration of C-O in C-O-C	Lignin

**Table 2:** The crystallinity index and crystal size of untreated basswood and delignified basswood.

	Untreated Basswood (Mean ± SD)	Delignified Basswood (Mean ± SD)
CrI	86.02 ± 0.64%	88.36 ± 0.56%
D_hkl_ (200)	30.37 ± 0.32 Å	26.22 ± 0.54 Å

**Table 3:** One-way ANOVA results of delignification time.

Source of Variation	Degree of Freedom	Sum of Squares	Mean Squares	F	F Critical	*p*-Value
Vin	6	153,102	25,517	49.89	2.35	0.00000
Vbetween	38	19,436	511			
Total	44	172,538				

**Table 4:** One-way ANOVA results of pressure.

Source of Variation	Degree of Freedom	Sum of Squares	Mean Squares	F	F Critical	*p*-Value
Vin	3	19,717	6572	13.84	3.07	0.00003
Vbetween	21	9974	475			
Total	24	29,691				

**Table 5:** One-way ANOVA results of moisture content.

Source of Variation	Degree of Freedom	Sum of Squares	Mean Squares	F	F Critical	*p*-Value
Vin	5	393,388	78,678	300.68	2.51	0.00000
Vbetween	32	8373	262			
Total	37	401,761				

**Table 6:** One-way ANOVA results of time of sealing.

Source of Variation	Degree of Freedom	Sum of Squares	Mean Squares	F	F Critical	*p*-Value
Vin	1	15,947	15,947	27.34	4.74	0.00021
Vbetween	12	6998	583			
Total	13	22,945				

## Data Availability

Data are contained within the article.
